# Supplementary figures and images for: METTL16 controls Kaposi’s sarcoma-associated herpesvirus replication by regulating S-adenosylmethionine cycle
Source: Cell Death Dis. 2023 Sep 6;14(9):591. doi: 10.1038/s41419-023-06121-3 (PMC10482891; doi:10.1038/s41419-023-06121-3)

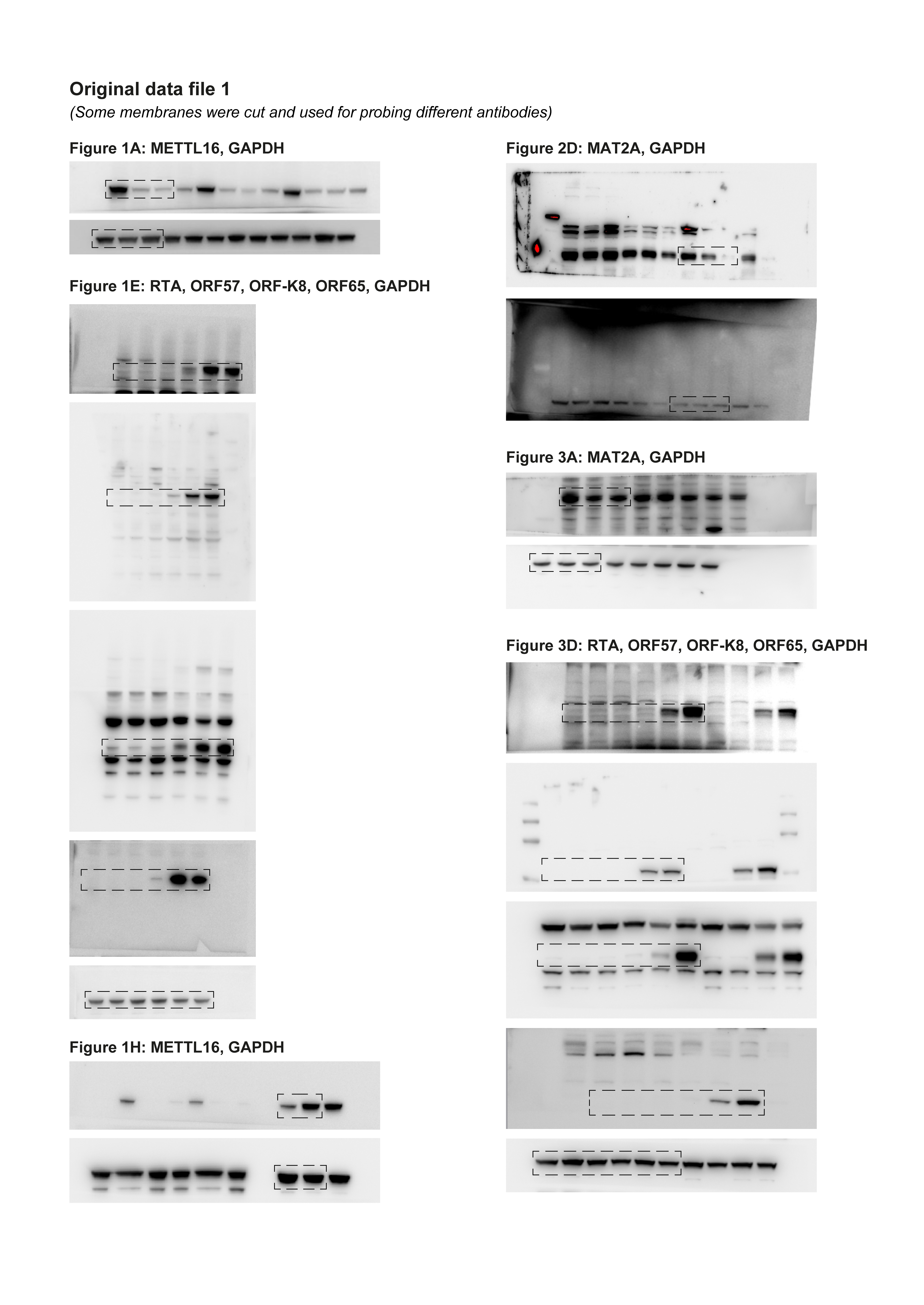

Supplement: Supplementary file 1 — Original Data File 1 [file 41419_2023_6121_MOESM1_ESM.tif]

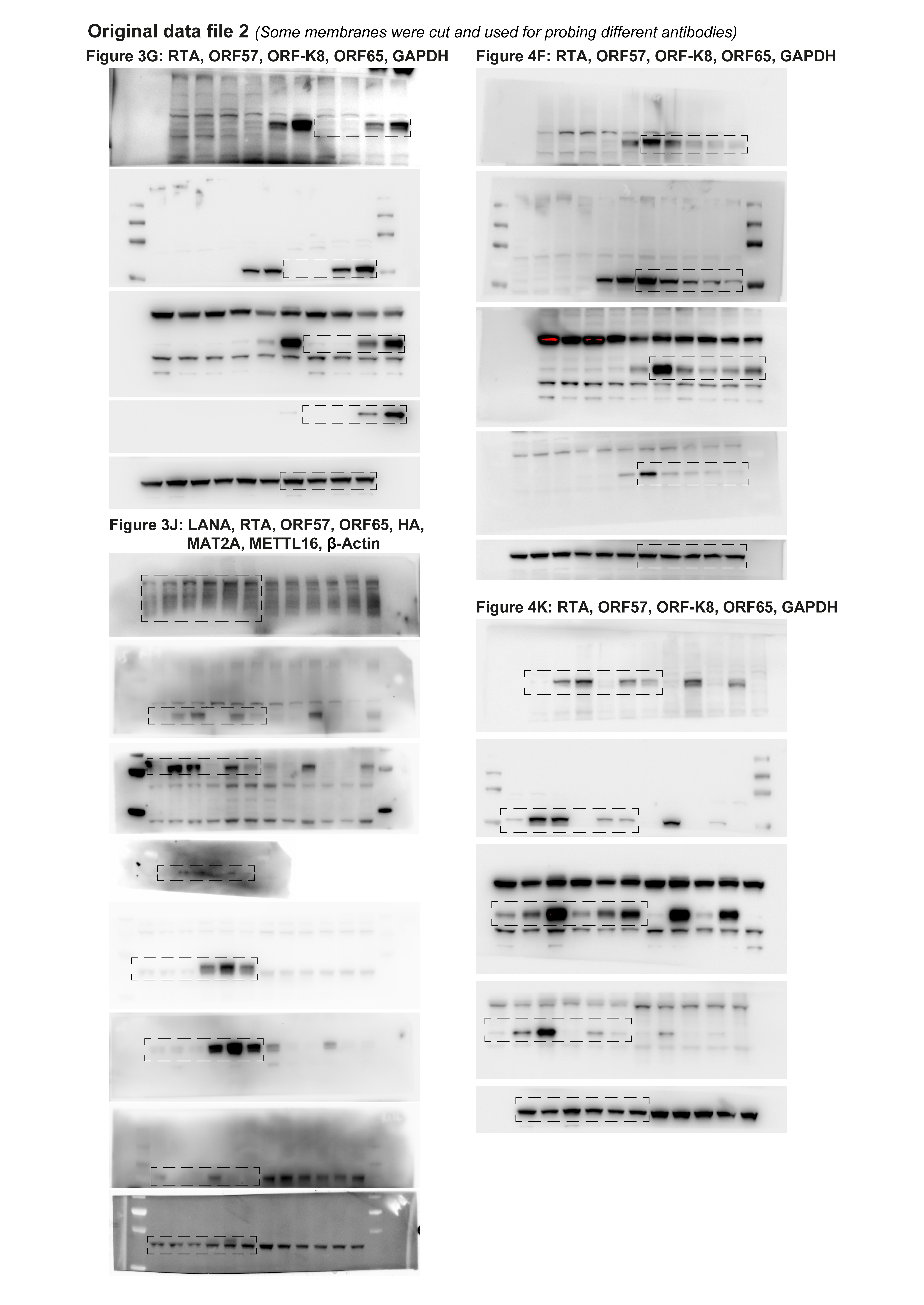

Supplement: Supplementary file 2 — Original Data File 2 [file 41419_2023_6121_MOESM2_ESM.tif]
